# Patient and Caregiver Experiences of Participating in Parkinson’s Disease Clinical Trials: A Systematic Review of Qualitative Studies

**DOI:** 10.1093/arclin/acab083

**Published:** 2021-10-20

**Authors:** Olivia Gorzynska, Katie McGoohan, Latha Velayudhan

**Affiliations:** Division of Academic Psychiatry, Department of Old Age Psychiatry, Institute of Psychiatry, Psychology and Neuroscience, King’s College London, London, UK; Division of Academic Psychiatry, Department of Old Age Psychiatry, Institute of Psychiatry, Psychology and Neuroscience, King’s College London, London, UK; Division of Academic Psychiatry, Department of Old Age Psychiatry, Institute of Psychiatry, Psychology and Neuroscience, King’s College London, London, UK

**Keywords:** Parkinson’s disease, Systematic review, Qualitative studies, Clinical trials, Caregivers

## Abstract

**Background:**

Older people experience multiple barriers to enrolment in clinical trials. Caregivers play an important role in supporting patients with Parkinson’s disease. Understanding the experiences of patients and caregivers who participate in trials is important to inform the design of future studies and identify problems with recruitment and retention.

**Objective:**

To systematically review and synthesize qualitative studies exploring the experiences of participating in clinical trials from the perspectives of patients with Parkinson’s disease and their caregivers.

**Methods:**

Two reviewers independently searched the following databases: MEDLINE, Embase, PsycInfo, Cochrane, and CINAHL. The reference lists of all selected papers were screened for additional studies. Articles meeting predefined eligibility criteria were included in the synthesis. Methodological quality of each study was assessed using the Critical Appraisal Skills Programme (CASP) Qualitative Checklist. Included study findings were synthesized using the principles of thematic analysis.

**Results:**

Eleven studies were included. Five key themes were identified: positive experiences of participating in research, assessment completion, motivators, enablers, and barriers. Positive experiences of participating in studies were linked to social interaction with other patients, building trust with the researchers, and expertise of the research team.

**Conclusions:**

This review supports literature highlighting the important role of caregivers in supporting patients with Parkinson’s disease. Future studies are needed to further examine their perspectives on participating in research.

## Introduction

Clinical trials are crucial for the development of novel treatments targeting the variety of symptoms in Parkinson’s disease (PD) and ultimately bringing us closer to a cure. Nevertheless, enrollment difficulties are frequently encountered by researchers, which leads to scientific problems of low statistical power and financial implications of higher study costs caused by prolonging trial duration to meet recruitment targets (Gul & Ali, 2010; Page & Persch, 2013).

Older people, who in vast majority constitute the PD patient population, experience multiple barriers that prevent their enrollment in clinical trials, such as comorbidities and cognitive impairment (Witham & McMurdo, 2007). Previous research suggests that key factors facilitating research enrollment for patients with neurological conditions include having information provided from a reliable source, adequate reimbursement, and the researchers’ attitude toward participants (Lindblad, Zingeser, & Sismanyazici-Navaie, 2011). On the other hand, transportation problems, frequency of visits, high cost, and lengthy and hard-to-comprehend information leaflets serve as barriers to participation (Lindblad et al., 2011; Locock & Smith, 2011).

Furthermore, when considering the factors influencing patient participation in PD clinical trials, it is important not to neglect the crucial role of caregivers. A caregiver can be defined as someone who looks after another person who needs care and support, and who is not paid to provide care (Bastawrous, 2013). The perception of research participation as an additional burden for the caregiver by other patient populations was identified as a barrier to study enrollment (Buss et al., 2008; Heckel, Gunn, & Livingston, 2018). Moreover, although older patients make autonomous decisions to participate in research, they often take into account opinions of those close to them (Witham & McMurdo, 2007).

Although randomized controlled trials (RCTs) are the “gold standard” in evaluating new treatments, qualitative research methods can address those questions that are not easily addressed by quantitative methods alone, by providing insights into the experiences of participants and caregivers (Gibson, Timlin, Curran, & Wattis, 2004). Using qualitative methods in clinical trials can carry numerous benefits, such as facilitating our understanding of the complexity of interventions, informing the design of future RCTs, and identifying problems with recruitment and retention (Featherstone & Donovan, 1998).

Although barriers and facilitators to research participation have been identified by previous research in other populations, to date there has been no synthesis of qualitative findings that can help us understand participant experiences among PD patients. This is therefore to our knowledge the first systematic review that aims to investigate PD patients’ and caregivers’ experiences of participating in clinical trials, explore what motivates PD patients to enroll in research, and identify what are the enablers and barriers to participation in clinical trials from the perspectives of PD patients and their caregivers.

## Materials and Methods

### Search Strategy

Five databases were searched for published studies by two independent reviewers: MEDLINE (via PubMed), Embase (via Ovid), PsycInfo (via Ovid), Cochrane, and CINAHL (via EBSCO) for the period from January 2000 to October 2020. The full-text search terms used to identify potential articles were “(Parkinson*) AND (qualitative OR interview) AND (trial)”. A full search strategy for PubMed is detailed in Appendix 1, (see Supplementary material online). The reference lists of all selected papers were screened for additional studies.

The review was not registered and the protocol was not prepared.

### Eligibility Criteria

This review considered intervention studies, such as clinical trials, feasibility studies, and mixed method studies, that involved qualitative data collection methods (e.g., semistructured or focus group interviews) and analysis to explore patient and caregiver experiences of taking part in trials. Qualitative studies that investigated this topic were also considered. Review articles were excluded. The population of interest consisted of patients with an established PD diagnosis (age 40–90 years) and/or their adult carers (18 years of age or older) of all genders and ethnicities. Articles were included if they were published in English. Studies were excluded if they did not report qualitative data (or the reviewers could not obtain additional reports directly from the authors), explored a phenomenon of interest that did not match the objectives of this review (e.g., focused on the effects of the intervention rather than participation), focused on the experiences of individuals with other conditions (e.g., dementia such as Alzheimer’s and PD dementia), or combined the experiences of individuals with PD and other conditions where experiences were not separated.

### Data Extraction and Quality Appraisal

Specific details such as the study participants, research aims, methodology, and findings relevant to the objectives of the review were extracted from included papers. For two papers (Kunkel et al., 2017; Paterson, Allen, Browning, Barlow, & Ewings, 2005), data were extracted from separate reports obtained directly from the authors. Eligible articles were critically assessed by two independent reviewers using the Critical Appraisal Skills Programme (CASP) Qualitative Checklist (Critical Appraisal Skills Programme, 2018). The purpose of this assessment was to evaluate the methodological quality of each study and to establish the extent to which a paper has addressed the possibility of bias. The tool assesses 10 criteria relevant to the quality of research, such as appropriateness of methodology and ethical considerations. Discrepancies were discussed between the reviewers and further discussed with senior researcher if needed for resolution. All studies were deemed high quality and no articles were excluded based on methodological appraisal. A summary of included articles with key limitations and comments from quality assessment is presented in Table 1 (see Appendix 2, Supplementary Table S1, for full appraisal).

**Table 1:** Summary of included studies

Article and country	Aims	Participants	Data collection and analysis method	Author-derived themes	Comments on quality appraisal and study limitations
Daley and colleagues (2015), UK	To investigate the experience of PD patients who received adherence therapy (AT)	10 PD patients who took part in an RCT of AT and 6 spouses/carers	Semistructured interviews, thematic analysis	Perceptions prior to AT; positive effects of AT; attributes of AT.	Transparent reporting of research methods; few negative experiences identified, potentially due to moderator bias (the therapist was also the interviewer, which hasn’t been critically examined in regard to potential bias and influence during data collection); small sample, but representative due to purposive sampling; sufficient data presented to support the findings; results well discussed in relation to research questions.
Khalil and colleagues (2017), Jordan	To explore cultural specifications, challenges, and enablers in participation in home-based exercise program in PD patients of Arab ethnicity who live in Jordan	16 PD patients who participated in a pilot study of a home-based DVD exercise and walking program	Interviews (not specified), thematic analysis	Enablers; personal challenges; cultural challenges.	Inconsistent transparency in reporting of methodology; researcher biases not explored; additional contextual details aid understanding of findings; some themes lack supporting quotes.
Kim and colleagues (2012), USA	To examine participants’ understanding and attitudes regarding sham surgery	90 participants from 3 sham surgery-controlled trials for PD	Semistructured interviews, content analysis	Understanding sham surgery research design, rationale, and procedures; attitudes toward sham surgery design; understanding and attitudes toward later offer of study intervention.	Reporting of methodology not transparent; researcher biases not discussed; qualitative findings reported in a quantitative manner; small number of illustrative quotes provided to support results; findings largely descriptive in nature and not presented in depth; times of interviews varied; missing data for interview questions.
Kim and colleagues (2015), USA	To examine whether subjects’ therapeutic motivation and own doctor functioning as researcher are associated with therapeutic misconception; to understand how research subjects make their research participation decisions	90 participants from 3 sham surgery-controlled trials for PD	Semistructured interviews, transcripts were coded and organized around responses to the following question: “What is your main reason for participating in the study?”	Motivation for participation: direct personal benefit; altruistic motivation; dual motivation (direct personal benefit and altruistic motivation); other.	Transparent reporting; researcher biases not discussed; qualitative findings presented in a quantitative manner; few supporting quotes included; findings descriptive in nature; interviews conducted at variable time points.
Kunkel and colleagues (2017), UK	To explore the experience of PD patients who participated in a randomized controlled feasibility trial of a ballroom dancing program	14 PD patients and their dance partners	Semistructured interviews, framework analysis	Background of participants; beginning and attendance; acceptability; challenges encountered in the learning process; outcomes of dancing; partner issues; participating in a research study; continuing with dancing.	Inconsistent transparency in reporting of methods; researcher biases not explored; results discussed only in relation to feasibility and no qualitative findings reported (detailed description of qualitative data collection, analysis and findings presented in a separate data report obtained directly from the author); findings in the report described in rich contextual detail with quotes provided in support; in some cases, data from caregivers included.
Lai and colleagues (2020), USA	To explore participants’ perceptions of intervention implementation processes and adherence	20 PD patients who participated in a mixed-method pilot study of Internet-exercise training	Semistructured interviews, thematic analysis	Positive program experiences; suggestions for improving the technology; challenges that affected exercise adherence; potential benefits of telehealth.	Most aspects of the methodology reported in detail; description of data analysis process lacks transparency; relationship between the researcher and participants critically examined (the interviewer was also a telecoach; this was identified as a potential cause of reluctancy of the participants to report negative feedback during interviews); description of qualitative findings brief and lacking depth; sufficient number of quotes presented to illustrate each theme; study sample potentially unrepresentative of the population.
Mammen and colleagues (2018), USA	To determine how patients and physicians perceive virtual visits and identify components contributing to positive and negative perceptions	149 PD patients who participated in an RCT of virtual house calls and 20 physicians	Qualitative survey (open-ended questions), content analysis	Personal benefits of the virtual visit; perceived quality of care; perceived quality of interpersonal engagement.	Most elements of the study methods reported clearly; researcher biases not discussed; analysis process described in detail; illustrative quotes presented to support the findings; contradictory data taken into account; study participants more familiar with the Internet than the population; survey responses potentially biased toward those who felt most strongly about the intervention; responses could not be explored further or contextualized.
O'Brien and colleagues (2008), Australia	To explore participants’ perceptions about community-based progressive resistance strengthening programme (PRST), motivators to begin and continue with the program, and facilitators and barriers to participation	12 PD patients who participated in an RCT of PRST	Semistructured interviews, thematic analysis	Motivators for participation in the PRST program were broader than physical outcomes; the outcomes were broader than just physical outcomes; the indicators of success for participants varied; the participants’ experience of a disease-specific exercise program was positive.	Study design and methods described in great detail; researcher background discussed; potential biases explored; numerous quotes presented in support of the themes; findings potentially not generalizable to people with PD who have more severe disabilities.
Paterson and colleagues (2005), UK	To explore the experience of massage therapy for people with PD from several perspectives; to examine whether relevant quality of life measures adequately reflect the experience and perceptions of patients receiving massage	Five PD patients who participated in a prospective longitudinal cohort pilot study of therapeutic massage	Semistructured interviews, content analysis	Living with PD and its treatment, and the experience of massage therapy; participant’s experience of questionnaire completion.	Transparent reporting of the methods; role of the researchers and potential biases not critically examined; no investigator triangulation for qualitative analysis; results described in full in a separate report; findings largely descriptive with rich contextual detail and focused on the individual; some supporting quotes present; findings on questionnaire completion mostly informed by researchers’ observations rather than participants’ voices; small sample size.
Rowsell and colleagues (2020), UK	To explore participants’ experiences of the intervention, its perceived impacts and facilitators and barriers to engagement	42 PD patients who participated in an RCT of home-delivered physiotherapy	Semistructured interviews, thematic analysis	Expectations of participants about the PDSAFE intervention; experiences and perceived impacts of the PDSAFE intervention; barriers and facilitators to participation in the PDSAFE program.	Detailed reporting of the design and methodology; reflexivity in reporting present; relationship between the interviewer and participants considered (the interviewer wasn’t previously involved in the intervention); investigator triangulation (two analysts) and peer checking utilized to enhance credibility of findings; extensive description of the results; numerous quotes presented to support the points made.
Sturkenboom and colleagues (2013), Netherlands	To explore perceptions of the intervention procedures and outcome	Patients with PD (*n* = 7), who participated in an RCT of occupational therapy, their primary caregivers (*n* = 7), and occupational therapists (*n* = 7)	Semistructured interviews, constant comparative method	Results; possible barriers and facilitators.	Inconsistent transparency in reporting; relationship between the interviewer and participants considered (interviews conducted by a research assistant not involved in the trial); potential biases not explored; brief and descriptive reporting of qualitative findings; illustrative quotes presented to support the derived themes; data from caregivers included.

### Data Synthesis

The extracted findings from eligible articles were synthesized using the principles of Braun and Clarke’s thematic analysis (Braun & Clarke, 2006). The reviewers familiarized themselves with the findings of each paper during full text screening. Using a deductive approach, the authors independently coded relevant data around predefined review objectives, which served as initial themes. Data were coded based on similarity in meaning to form the basis of recurring patterns. When all data have been coded, the codes were organized into themes and subthemes centered around patient and caregiver experiences of participating in trials, motivators for enrollment, and facilitators and barriers to participation. Supporting participant quotes were then collated to illustrate each theme. Data extracts for each theme were reviewed to confirm they followed a coherent pattern. All themes were also reviewed in relation to all extracted data to ensure the relationships between themes reflect the meaning of the dataset as a whole. Concise theme names were then devised to capture the essence of their conveyed content. As a last step, developed themes were discussed among the two reviewers and any discrepancies resolved following discussions with a senior researcher. This process of peer review was utilized to minimize researcher bias and enhance the reliability of data synthesis. Although the reviewers generated different theme names, the meaning of each theme was consistent between them. Theme names were compared among the reviewers and the most suitable was chosen.

## Results

### Literature Search and Characteristics of Included Studies

**Fig. 1:**
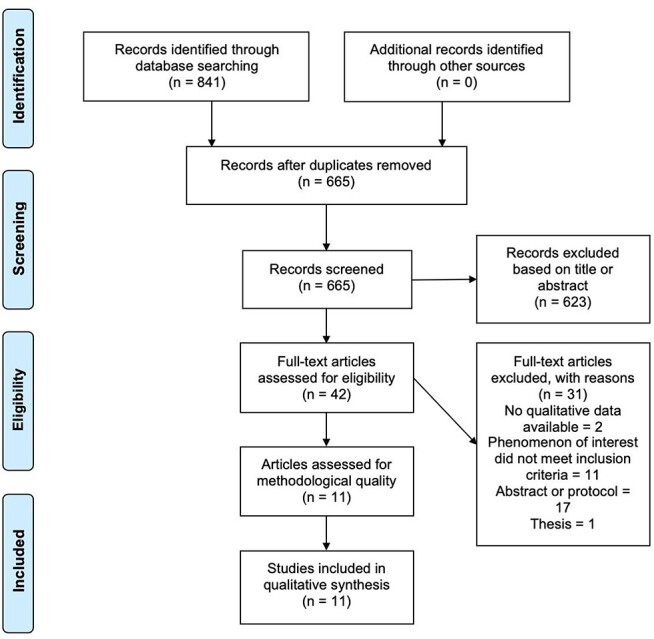
Flowchart of article selection process.

A comprehensive search of the databases yielded a total of 841 results, including duplicates. No additional articles were found after reviewing the reference lists of relevant studies. Screening and removing duplicates brought the number of articles down to 665. Following exclusion by the titles and abstracts, full texts of 42 remaining articles were retrieved for further review, of which 11 studies fulfilled the inclusion criteria (Fig. 1 PRISMA flow diagram) (Moher, Liberati, Tetzlaff, & Altman, 2009).

Characteristics of included papers are summarized in Table 1. The 11 papers included in this systematic review reported data on the experiences and attitudes of PD patients and their caregivers in relation to the interventions, their motivators for participation, and perceived facilitators and challenges to engagement.

The studies utilized qualitative data collection methods as a part of RCTs (Daley, Deane, Gray, Hill, & Myint, 2015; Kim et al., 2012, 2015; Kunkel et al., 2017; Mammen et al., 2018; O'Brien, Dodd, & Bilney, 2008; Rowsell et al., 2020; Sturkenboom et al., 2013), mixed-method pilot studies (Khalil et al., 2017; Lai et al., 2020), and a prospective longitudinal cohort pilot study (Paterson et al., 2005).

The trials were carried out in the UK (Daley et al., 2015; Kunkel et al., 2017; Paterson et al., 2005; Rowsell et al., 2020), Jordan (Khalil et al., 2017), the USA (Kim et al., 2012, 2015; Lai et al., 2020; Mammen et al., 2018), Australia (O'Brien et al., 2008), and the Netherlands (Sturkenboom et al., 2013). Sample sizes varied between 5 and 149 participants with mild to moderate disease severity and a mix of genders. Two studies included the participants’ caregivers (Daley et al., 2015; Sturkenboom et al., 2013), and one study (Kunkel et al., 2017) included the patients’ dance partners, who were their spouses, friends, or volunteers previously unknown to them.

Data were collected mostly through semistructured, focus group, or otherwise unspecified interviews. One study (Mammen et al., 2018) used an open-ended survey. Data analysis approaches included thematic analysis, constant comparison, and framework analysis.

None of the trials investigated pharmacological interventions. Two studies (which utilized the same sample) explored attitudes toward and motivators for participation in sham surgery-controlled trials (Kim et al., 2012, 2015). Four articles examined the experiences of PD patients who took part in various physical exercise interventions, such as ballroom dancing (Kunkel et al., 2017), home exercise (Khalil et al., 2017; Lai et al., 2020), and resistance training (O'Brien et al., 2008). Four papers investigated participants’ perspectives on different therapeutic approaches, namely adherence therapy (Daley et al., 2015), massage therapy (Paterson et al., 2005), home-delivered physiotherapy (Rowsell et al., 2020), and occupational therapy (Sturkenboom et al., 2013). One article focused on virtual physician visits (Mammen et al., 2018).

### Themes

Five themes and thirty-one subthemes were identified by review authors. The themes were organized in regard to the three study objectives. The main themes, subthemes, and supporting quotes are presented in Table 2.

**Table 2:** Main themes, subthemes, and supporting quotes organized by review objectives

Themes	Subthemes	Supporting quotes
***Objective 1: Investigate PD patients’ and caregivers’ experiences of participating in clinical trials***		
1a. Positive experiences of participating in research	Benefits of a positive and equal relationship with the person delivering the intervention who is knowledgeable about PD	Participants explained that the characteristics of the person delivering the intervention could have a significant impact on their experience. Building trust and engagement with patients could be facilitated through qualities such as expert knowledge of PD, honesty, patience, and empathy: • “Because you had an understanding of PD I was halfway there. There were so many issues I had that we spoke about. I’ve not been able to do that with others.” (Participant, Daley et al., 2015) • “It was welcoming not having to justify some of my actions. I felt I could say anything without you judging.” (Participant, Daley et al., 2015) • Partner: “They were very sympathetic and very patient with us all.” • Patient: “Yes, they needed to be didn’t they! (. . .) the whole team was very, very good” (Kunkel et al., 2017 report) • “It gives a basis of trust and contact. You are being heard. So because of that I think I become opener, because she listens.” (Patient, Sturkenboom et al., 2013) • “I felt comfortable telling you my fears. I was able to say the simplest things and you put me at ease.” (Participant, Daley et al., 2015) • “Attention for me as a person, looking at the situation, adjusting interventions to the situation, practical, thinking creatively to find what fits best in that situation.” (Patient, Sturkenboom et al., 2013) • “Yes, there was a click, so then it becomes a lot easier.” (Caregiver, Sturkenboom et al., 2013)
	Sense of achievement, increased confidence, and mental well-being	Patients and caregivers discussed that enjoyment of participation was linked to a sense of achievement, which contributed to increased morale and self-confidence. The benefits of interventions included improvements in both physical and mental well-being: • “I mean I didn’t have any expectations. . . but both my morale and confidence were lifted as I came to the end of each class and I was still up and running.” (Participant, Kunkel et al., 2017 report) • “As I said, you know they did, really did stretch us. So that surprised me, because I thought we’d be doing pretty basic things, but in the end you were doing spins, and gee, it was incredible.” (Spouse of patient, Kunkel et al., 2017 report) • “Well I think, I think it has done me some good. I mean certainly in terms of morale, you know, that one was able to keep up the pace, and that it was something that turned out to be an enjoyable experience.” (Participant, Kunkel et al., 2017 report) • “Marvellous. My well-being, you know overall well-being been really great. I’ve gained confidence. I’ve been really good. Doctor just can’t believe it.” (Participant, Paterson et al., 2005 report) • “Mentally that had a very positive effect. Because you [herself] get grip of the situation and the feeling of yes he [patient] can do it, although just in another way.” (Caregiver, Sturkenboom et al., 2013) • “The exercise program has affected me both mentally and physically before I got to know you and was introduced to this program I used to lock myself away at home fear of falling was a big issue. I was therefore not moving. . . Now my mobility has improved dramatically. . . I feel as If I have regained big chunk of my life.” (Participant, Khalil et al., 2017)
	Rewarding social interaction with other participants	Patients with PD enjoyed social interaction with other participants: • “Interviewer: What was it in particular do you think that you liked about it? PWPD [Person with Parkinson's disease]: Well it's another day to go somewhere and to meet people, and we all talked to each other and we were quite, you know a good group. So it was really nice. Interviewer: So was that social aspect of it quite important to you? PWPD: Oh yes. Yes, because I like anything like that because there's nothing worse than sticking indoors and not doing anything or going anywhere, because you tend to vegetate then don't you? So I'm out all the time. I'm always out somewhere.” (Kunkel et al. (Kunkel et al., 2017) report) • “No. I did not notice any changes in the strength of my arms or legs. . .I enjoyed being part of the group and going out to do something for myself.” (Participant, O'Brien et al., 2008) • “I did enjoy the social contact with the other people who were involved in the programme, and I just felt that within myself that it would have to be doing me good. Have to be helping me, so it’s just positive outlook. The contact with the other people’s really good and I’ll be keeping in touch with those people.” (Participant, O'Brien et al., 2008) • “Partner: Well I mean we’ve enjoyed the social aspect as well as the actual exercise as well.” (Kunkel et al., 2017 report) • “We got to know them, we were friends with them and everything and what I noticed a lot of them were dreading it finishing because they enjoyed the company and the outing and they were wondering they were all saying well it’s not long enough, we’re all starting to talk in the last sort of two or three weeks you know. And they were all saying oh it’s not long enough. . . Because as I say, we were all getting quite friendly and quite a nice little group you know.” (Participant, Kunkel et al., 2017 report) • “I haven’t noticed anything physically; maybe just a marginal improvement in the strength of my arms. With regard to my legs, I don’t think there has been much change at all, but mentally I think, oh I don’t know, I think just being involved with other people sort of, even at this level, helps me a bit.” (Participant, O'Brien et al., 2008) • “I suppose living on my own, it’s just nice to get out with other people, even if it’s just a marginal contact, and I was able to talk with a neighbour who gave me a lift into the classes, and that was just an extra contact each time.” (Participant, O'Brien et al., 2008) • “We had a good social feeling amongst the people. . . we’ve met new people, and that in itself was probably worthwhile, as well as the physical side.” (Participant, O'Brien et al., 2008)
	Benefits of meeting other people with a similar condition	Interacting with other participants was often seen as a beneficial opportunity to exchange PD-specific information: • “[We] exchanged thoughts and notes that we had we all seem to have been on the Internet at various stages, gathering information, and it was very helpful.” (Participant, O'Brien et al., 2008) • “It’s just that contact with other people and you know, if anything little comes up, even you know just generally people talking amongst themselves about medication, sorts of effects that they are having and that type of thing, yes, just really having no contact with anybody else that’s afflicted by Parkinson’s is good to have that sort of contact, so because it’s tailored to that type of group.” (Participant, O'Brien et al., 2008) • “Well I’ve learned that there’s so many people in the same boat as I am. And I’ve learned to take it a bit more quietly and be a bit more laid back about it.” (Participant, O'Brien et al., 2008) • “The people I was with were much about the same state as I were as they weren’t sort of further down the track with Parkinson’s. . . there wasn’t a lot of difference between us. If I’d seen someone as we came in. . . [who] had the disease a lot longer I might have been a bit more apprehensive then I suppose, but I think because I was in with other people whose condition was very similar to mine, it was quite good.” (Participant, O'Brien et al., 2008) • “PWPD: It was really, quite nice to feel you were back in the real world doing stuff that other people did, you know, dancing and classes. Partner: Yes, and seeing how other people with Parkinson’s coped with it and you know it’s so varied and affects people in such different ways, that it was interesting to see how couples coped.” (Kunkel et al., 2017 report)
	Increased understanding of condition	Patients indicated appreciation for interventions that allowed them to learn more about PD and understand it is personal to everyone: • “Best thing that came out of it is that I found out more about my Parkinson’s as a personal thing to me and I found out that it didn’t mean that the person I saw walking past with Parkinson’s was one who was like I am going to be, because it is all different, so don’t be sitting here thinking ‘Oh god, I am going to end up like that’ because you might not, and I found it has definitely made me better.” (Participant, Rowsell et al., 2020) • “…the insight. Of what makes up the strain and how I can better influence that, how I can better balance it. That helped me a lot.” (Patient, Sturkenboom et al., 2013)
1b. Assessment completion	General experiences of questionnaire completion	The participants reported no major issues with the questionnaires: • “Richard couldn’t remember the questionnaires in any detail, but didn’t feel they had been a problem to complete. (. . .) He didn’t have any criticisms of the questionnaires generally, except it would have been easier if the previous scores were available for comparison.” (Author, Paterson et al., 2005 report) • “Jane [care partner] thought it was hard to indicate a change, without knowing previous scores, and would have preferred a ‘bit better’ type response choice.” (Author, Paterson et al., 2005 report) • “With guidance Terry quickly grasped the MYMOP and PDQ-39 questions, and there appeared no major problems.” (Author, Paterson et al., 2005 report) • MYMOP: “The choice of symptoms appeared straightforward and remained appropriate at the end of the study, and most people were able score the severity without difficulty.” (Author, Paterson et al., 2005 report) • MYMOP: “The choice of an activity was a little more problematic, as several people chose activities that they had not done for some time and had no expectation of doing, such as dancing and table-tennis. It had proved difficult to find other alternatives, especially as ‘walking’ was often chosen as symptom 1.” (Author, Paterson et al., 2005 report) • MYMOP: “Participants were able to score their wellbeing without difficulty and conceptualised it as ‘the way I feel within me. . .happy..’ or ‘how I feel within myself’ or they related it closely to emotional health.” (Author, Paterson et al., 2005 report) • PDQ-39: “Generally people appreciated the content of this questionnaire and considered it covered most of their problems, and were not put off by its length (with someone helping them).” (Author, Paterson et al., 2005 report) • PDQ-39: “The sections on emotional health, social support and stigma gave rise to few problems, although one person commented that having the spouse involved in helping complete the questionnaires may have affected the responses here.” (Author, Paterson et al., 2005 report) • PDQ-39: “The communication questions appeared straightforward (. . .)”(Author, Paterson et al., 2005 report) • “(. . .) no one found the questionnaires too burdensome, and some people were quite happy with them.” (Author, Paterson et al., 2005 report)
	Reflecting participants’ experiences (PDQ-39)	It has been observed that the questionnaires had a limited ability to reflect patients’ experiences accurately: • “Choice of response was easy when something was very bad or very good, and most difficult ‘when it comes to, like dressing and that well if you can do part of it and not all of it” (Care partner, Paterson et al., 2005 report) • “Activities of daily living were difficult to score because he needed help with only some aspects of washing and dressing, and had adapted a few things to make it easier.” (Author, Paterson et al., 2005 report) • “The sections on mobility and activities of daily living asked mainly about function, and gave rise to the following problems: (a) Choosing a response option when the activity can be done in part but not completely, such as looking after your home (able to do housework and cooking but not DIY); dressing (able to dress except for socks and shoes); and washing (able to wash and shower but not bath). The fact that the response options asked ‘how often’ rather than’ to what extent’ made this more difficult, and respondents generally just picked a middle option. (b) Knowing how to reflect that they had either adapted their life, or had enlisted help, with an activity so that it was now not much of a practical problem, yet they were distressed at having to make the changes. For example neighbours helping with the shopping, family helping with things around the house, wearing clothes that are easy to put on, allowing a lot of time to complete a task. (c) Needing a ‘not applicable’ option for activities that are not part of their normal life, for example two men were married to women who had always done most of the ‘looking after your home’ activities.” (Author, Paterson et al., 2005 report) • “The questions on cognition gave rise to the following problems: (a) Two people had difficulties responding to ‘Had distressing dreams or hallucinations’ as they had hallucinations but they were not distressing. (b) The wife of one of these thought that confusion needed to be scored separately. It may be a function of hallucinations and of poor memory but was the important outcome of these.” (Author, Paterson et al., 2005 report) • “The responses to the mobility questions were good because her neighbours helped with shopping and her son helped with DIY. However they may not reflect her feelings around mobility, such as her distress at not being able to do DIY jobs herself anymore, not being able to enjoy walking for pleasure, and not feeling confident on steps etc.” (Author, Paterson et al., 2005 report) • “The cognition questions were the least satisfactory, as he had hallucinations but they were not distressing to him. On the other hand they caused confusion that was a problem.” (Author, Paterson et al., 2005 report)
	Being unsure of what constitutes an accurate response on questionnaires	Participants expressed having difficulties choosing an accurate response on self-report questionnaires due to the problematic wording of the answer options: • “Bill: I didn’t want to put ‘never’, I mean you might want (indistinct phrase) C: So you thought that was a bit risky putting ‘never’ Bill: Yes that’s right, that’s why I put. . . C: Because you mean you might, in the future something might happen Bill: that’s right. . . It’s better to be on the side of caution” (Paterson et al., 2005 report) • “I haven’t gone onto nought because uh I don’t really know what that means. . . how good is, how good is ‘as good as it can be’? That’s why I, that’s why I stayed on the one.” (Participant, Paterson et al., 2005 report) • “I just wondered if I had given the right answers. . . Because you know you’ve got this like occasionally, or sometimes, and you think well, what’s occasionally and what’s sometimes? Surely the two are the same?” (Alice, Paterson et al., 2005 report) • “It appeared that on the whole people accommodated for these difficulties by making common-sense and appropriate adjustments to their scores. The interviewer’s observation was that, especially on the functional questions, the response options of frequency of a problem did not often make logical sense (mostly people had some difficulty all the time, rather than complete difficulty some part of the time). However this was not raised by any of the participants, nor was it taken up if the interviewer introduced it into the discussion. It would seem that people were basically able to use the response options as gradations of severity. It may be, however, that this will result in most responses being ‘occasionally’ and ‘sometimes’, and that this will reduce sensitivity to change.” (Author, Paterson et al., 2005 report)
	Requiring assistance	Most patients required some assistance with completing self-report questionnaires: • “In view of her poor sight and concentration, Alice appreciated MB reading out the questions for her, but stressed that the answers were hers alone.” (Author, Paterson et al., 2005 report) • “Oh yes, yes, she more or less asked Tom you know, but as I said you know I had to give her, well just translate it really.” (Spouse of participant, Paterson et al., 2005 report)
	Variability of symptoms	It has been observed that the variability of PD symptoms could make assessments difficult: • “The trouble with assessments is that, and I mean we’ve been very fortunate that R hasn’t had a reaction, but if we did get a dose failure just before an assessment, then that would give you a very distorted impression.” (Spouse of participant, Kunkel et al., 2017 report) • “And if you can get the funding, if the feasibility study shows even an inkling that it might be having an effect then you should do a follow-up, but I think you need, this . . . You need a large sample because of the innate variability within a person from day-to-day. I know on my follow up assessments with [research assistant] I wasn’t feeling particularly right so my turning was a lot more lame than on the previous time. But I think you should go for it and follow it up.” (Participant, Kunkel et al., 2017 report) • “Alice’s symptoms varied a lot from day to day, which made averaging out to one score difficult, and she thought she probably thought more of the last few days than the last week (MYMOP) or the last month (PDQ-39).” (Author, Paterson et al., 2005 report)
	Distinguishing symptoms of PD from other issues	Some patients highlighted having difficulties distinguishing the effects of PD from other problems when completing questionnaires: • “(…) for Alice household activities were hampered by a combination of stiff clumsy fingers (from PD) and poor sight.” (Author, Paterson et al., 2005 report) • “Her health and functional ability was affected by a combination of health problems, of which PD was only one. In completing the PDQ-39 she did not attempt, nor would she have been able to, to score just for the PD, so her scores also reflect her depression, arthritis pains etc.” (Author, Paterson et al., 2005 report) • “Bodily discomfort includes cramps and pain, but she thinks these may be mainly due to arthritis.” (Author, Paterson et al., 2005 report) • Several people found it difficult to know if their cognition deficits were due to PD or to age. (Author, Paterson et al., 2005 report) • “The communication questions appeared straightforward, but the bodily discomfort questions were a problem: Difficult to distinguish minor ache and pains of PD from arthritis and minor injuries and several people had occasional night cramps that they thought pretty normal for their age. This section appeared to be given scores that often reflected minor, or non-PD, conditions.” (Author, Paterson et al., 2005 report)
***Objective 2: Explore what motivates PD patients to enroll in clinical trials***		
2a. Motivators	Direct health benefit from the intervention	Participants identified deriving health benefits, such as improving symptoms or slowing down disease progression, as a key motivator for participation: • “My biggest motivation? I guess my biggest motivation is I would like to get better.” (Participant, Kim et al., 2015) • “PWPD: Well I think you grasp at straws, you know you want to do anything you can. Anything that came up I think we’d do wouldn’t we? Partner: Mm.” (Kunkel et al., 2017 report) • “Just to. . . slow down the progress of the, you know, deterioration of the limbs.” (Participant, O'Brien et al., 2008) • “But um, its really a case of hoping things will not get any worse than they are now” (Participant, Paterson et al., 2005 report) • “Yes, so, that’s really why I put my name down for it, because I thought, well anything that can help me keep going as long as possible, because I have got one daughter in [place] and one daughter in [place], so, and no daughters in England [laughter] and they are not likely to be coming back, so I want to keep going.” (Participant, Rowsell et al., 2020) • “I [Interviewer]: What is your understanding of what might happen when the physiotherapist comes to see you tomorrow and over the course of the next few months when you are working with the physio? P [Participant]: Now you’ve frightened me (laughs) . . . I haven’t really thought about it sufficiently. I’m hoping that they will give me some useful exercises that will deal with, well the balance, now the question in my mind, is whether they will go on from that and talk about also posture, which is something I desperately need and general muscle tone I suppose, so I’m sort of hoping that they will cover all three things.” (Rowsell et al., 2020)
	Altruistic motivation	Participants reported altruistic motivators for enrolling in trials, such as contributing to research and benefiting those who might experience PD in the future: • “No, I just . . . I just felt I should do something to help the next guy down the road.” (Participant, Kim et al., 2015) • “[I] was keen to try and assist the programme as a participant for the sake of future generations of PD sufferers.” (Participant, O'Brien et al., 2008) • “I don’t know how I got involved in it quite honestly. I think, well someone, someone said would you like to get involved in this study? You’ve probably seen this literature that came from [principal investigator]. I had the information that it took place twice a week, and I thought I’d go along and see if I could help in any way.” (Participant, Kunkel et al., 2017 report)
	Deriving health benefits and helping research	Some participants were motivated by both the desire to improve their symptoms and contribute to research: • “Well, an improvement in my condition. . .And, you know, to help further the research in Parkinson’s.” (Participant, Kim et al., 2015)
	Encouragement from others	Patients explained that other people, such as friends, family, and doctors, could play a role in encouraging participation: • “On hearing about the study from a researcher who came to PD support group, one PWPD had felt uncertain: “I thought this is a bit weird, I don’t think I ought to do this.’ At a subsequent meeting another speaker talked about the NIHR research promotion campaign ‘Get Involved’. This made him re-consider and discuss with his friends and family who encouraged him: “. . .so I thought goodness me, alright, so I did it. I went in for it.” (Kunkel et al., 2017 report) • “I’ve had some major surgery last year, and I thought that because I’m still recovering from that I may not be able to take part, and it was only through my own physiotherapist ringing me up and contacting me saying that she felt it would be good for me that I decided to go ahead with it. So the physiotherapist contacted my doctor, and he stated that yes, it would be okay if I do that, providing I’m careful with any exercises that might involve the neck. . .but he was quite happy for me to do it and my physiotherapist was quite happy so I agreed to go ahead and do it.” (Participant, O'Brien et al., 2008) • “And we are like “if they want to help you, you have to go for it”. (Caregiver, Sturkenboom et al., 2013) • “So what made you decide that you would take part in this dancing programme? Partner: I beat him! PWPD: I was coerced into it. Right. So [to partner] you thought it would be a good idea then? Partner: Yes. PWPD: Well a friend of ours saw it in The Echo, and cut it out, so you followed it up didn’t you? Partner: Yes. Well I had to follow it up with [husband]‘s permission obviously [laughing] Yes, I just felt he was in need of something to be a little bit more fluid. . . PWPD: Yes.” (Kunkel et al., 2017 report)
	Increased social interactions and to keep busy	Patients discussed enriching their life through social interactions and regular activities as a motivator for participation: • “Well somebody came to talk at our Parkinson’s coffee morning, and it sounded interesting and I quite fancied having a routine twice a week, a trip out . . . and meeting people, socialising, because although talking is problematic and tiring I enjoy it very much. So that was the opportunity to get out, you know get out and meet people, as well as producing a print on a graph which hopefully it’ll be a bit more than that. . . prints on several graphs.” (Participant, Kunkel et al., 2017 report) • “The actual going out and being with other people might be of some advantage.” (Participant, O'Brien et al., 2008) • “Well I just like any activity. I just like to keep busy, because it’s so good for the condition to keep busy.” (Participant, Kunkel et al., 2017 report)
	Interest in the intervention	Some participants expressed they decided to take part in research due to their interest in the intervention: • “The main motivation was just to find out more about strength training” (Participant, O'Brien et al., 2008) • “Oh I am pleased actually because I am never quite sure what exercises are beneficial and what are not. I mean I used to go to a Pilates class and I was beginning to find it really hard to do and I wasn’t sure if I should keep going and sort of push through it, even if it was hurting, or whether that was telling me I ought to stop, I don’t know. So I am really pleased to speak to somebody and have some exercises that are actually tailored to suit the condition.” (Participant, Rowsell et al., 2020) • “Well I thought, I thought it would be fun to do some dancing for a change, to go to a dance class.” (Participant, Kunkel et al., 2017 report) • “So when I got invited into it I just thought it was an opportunity, so a bit of fun really. Yeah, I just thought it was something different you know. And I enjoy dancing, I like music, so. . .” (Participant, Kunkel et al., 2017 report)
***Objective 3: Identify what are the enablers and barriers to participation in clinical trials from the perspectives of PD patients and their caregivers***		
3a. Enablers	Continuous monitoring and support	Patients identified continuous supervision and a supportive relationship with the therapist as factors important for adherence and building self-efficacy: • “Also having the physiotherapist visiting regularly; that gives you something to aim for, like, when you know that she is coming next week and you want to make sure that everything is up to date and that you are doing the exercises properly, so having that sort of check and balance if you like helps to keep and it ran very smoothly.” (Participant, Rowsell et al., 2020) • “Knowing he [the telecoach] was there watching, knowing he was gonna be there on the other side, you know, expecting me to do it. He wasn’t mean or anything like that, but he was encouraging, ‘Come on you can do it, you can do it.’ Made me want to. So I didn’t want to disappoint him.” (Participant, Lai et al., 2020) • “(. . .) we setup times and it gave me accountability to do it, and the monitoring helped in that accountability.” (Participant, Lai et al., 2020) • “I gained more confidence than I had [before the programme]. You [the telecoach] got me started on it, and I built more confidence after that. Confidence is what it boils down to. Exercise more, I get more confidence than I had.” (Participant, Lai et al., 2020) • “It was nice knowin’ that it ya’ll could watch what I was doin.” (Participant, Lai et al., 2020) • “I really prefer doing the exercises at home. . . the sessions with therapist were very important to know what I am supposed to do and to build confidence.” (Participant, Khalil et al., 2017) • “For the first time ever I felt that someone was truly taking care of me that was the best piece of the intervention. It made me feel in turn that I should take care of myself by committing to the exercise program. The therapist was an excellent motivator.” (Participant, Khalil et al., 2017)
	Positive attitudes toward study design	Positive attitudes toward study design could be a facilitator to participation: • “All good studies should be done that way” (Participant, Kim et al., 2012) • “[T]he fact that there was a placebo and it was double-blind. . .it did make me more confident that. . . [i]t’s a professional study as opposed to nonprofessional” (Participant, Kim et al., 2012)
	Openness to the intervention	Openness toward the intervention and its outcomes could serve as facilitators to adherence: • “So let it go, we’ll see what comes. That in itself I found quite pleasant.” (Caregiver, Sturkenboom et al., 2013)
	Caregiver and family support	Support and encouragement from the patient’s family members, spouse, or caregiver were perceived as important factors influencing adherence: • “For early stage participants, encouragement provided by family members was perceived to be important for initial adoption and for continuation: “The family encouragement was very important for me to take this step and start the exercise program with you.” (Participant, Khalil et al., 2017) • “Interviewer: How was that when she (i.e., the physiotherapist) wasn’t here? How was it doing the exercises? Participant: Fine. I: Were you doing them with (partner)? Partner: Yes, we had our little routine every morning, dancing around in the kitchen. I: Brilliant. (laughter)” (Rowsell et al., 2020) • “That [involvement in intervention] I found not more than normal. You are husband and wife. And especially these sort of things you have to do together.” (Caregiver, Sturkenboom et al., 2013) • “The presence of a spouse/carer appeared helpful, particularly where they had an active role in management of medication: “There was a lot for me as the carer because you opened my eyes to many things which I didn’t think were important.” (Spouse of participant, Daley et al., 2015) • “Also for her [caregiver] process, I think. She has to start realize as well what it [Parkinson’s disease] all involves. We both don’t know this.” (Patient, Sturkenboom et al., 2013)
	Clear and easy-to-follow interventions	Straightforward instructions and easy-to-follow interventions aided adherence: • “The DVD was simple and easy to follow its use at home was a strong motivator to continue doing the exercises.” (Participant, Khalil et al., 2017) • “It was easy enough to operate and wasn’t obtrusive. The software was straightforward.” (Participant, Lai et al., 2020)
	Benefits of at-home interventions	The convenience of at-home interventions aided adherence: • “The big advantage is I didn’t have to go anywhere. I didn’t have to get in the car and drive to a gym or somewhere else (. . .)” (Participant, Lai et al., 2020) • “It [the programme] is very convenient. In that you get to choose your times. You don’t have to leave home. It was also handled in a very professional way.” (Participant, Lai et al., 2020) • “I like the openness of the program[me], you can do it when you want to do it. It makes you responsible for your own exercise.” (Participant, Lai et al., 2020) • “Very convenient compared to 3.5 hour drive each way! Less expensive.” (Participant, Mammen et al., 2018) • “In the hospital you are in a theoretical situation, while my problems are here [at home]. So then she can better see what it looks like here and how we can adapt things than there [in hospital].” (Patient, Sturkenboom et al., 2013) • “I found that [treatment at home] real good. . . I believe that there you can pick up certain things best.” (Caregiver, Sturkenboom et al., 2013) • “The way it [AT] was presented made a hell of an impact. You can’t judge someone and their reactions over the phone.” (Participant, Daley et al., 2015) • “I liked the convenience of it [the programme]. Being able to do it in the home and not have to drive somewhere like a gym.” (Participant, Lai et al., 2020)
	Transportation arrangements	Participants valued travel arrangements and reimbursement: • “As you know I come from a distance and my participation would have been impossible without covering the transportation costs that was really important aspect.” (Participant, Khalil et al., 2017) • “The fact that there was easy parking there was a distinct advantage, particularly if people had a longer distance to travel.” (Participant, Kunkel et al., 2017 report) • “It couldn’t be better. A taxi came. I had a problem getting him to come at the right time. [To start with the taxi came] quite a lot too early, and then that was changed to five minutes late. Well I thought five minutes late is better than half an hour early, so I left it, but [research assistant] organised it and she was terrific and she ruled us with a gentle hand.” (Participant, Kunkel et al., 2017 report)
3b. Barriers	Denial of PD diagnosis	Denial or uncertainty of PD diagnosis was perceived as barriers to adherence: • “I did not do the exercises because I am still not convinced I have PD. . . I have this dilemma . . . I am really not convinced that I have PD . . . next week I will be seeing another neurologist to discuss my case.” (Participant, Khalil et al., 2017)
	Embarrassment or secrecy toward diagnosis due to cultural stigma	• “PD is a big secret in my life. No one apart from one very close friend knows about it. Even my wife. If I bring the DVD to home and start exercising they will start to ask the questions . . . basically it hurts but I still do not want them to know about it” (Participant, Khalil et al., 2017) • “At home [Jordan] I have the fear that my sons will comment on this I am trying to avoid this. I did though all the sessions with the therapist in the clinic but did not do the sessions at home.” (Participant, Khalil et al., 2017)
	Negative attitudes toward trial design	For some participants, negative perceptions about the trial design was a barrier to participation: • “[T]hat part of it is really a turn-off, a real big one” “I had to think about that for a little bit” “I was discouraged” (Participants, Kim et al., 2012)
	Issues with technology	Technical issues were a source of frustration and could negatively affect adherence: • “I couldn’t get the equipment to respond the way I wanted it to. . . It was frustrating trying to get the tech to work. After my expectations floundered, I just gave up.” (Participant, Lai et al., 2020) • “There were tech issues with the microphone, which meant we had to be on the phone and computer at the same time. Very frustrating. Otherwise things were fine.” (Participant, Mammen et al., 2018) • “The bad things I’d have to say about it is the WiFi was cutting out most of the time.” (Participant, Lai et al., 2020) • “Technology required a learning curve: “It took me a couple of weeks if not more, to figure out what I was doing. Or know how it operated.” (Participant, Lai et al., 2020)
	Not enough support	Participants identified the need for practical assistance and verbal support: • “It was just a little difficult to get setup. . . It would have helped to have someone for at least two times to be sure I was doing things correctly or not.” (Participant, Lai et al., 2020) • “It’s going to require people with Parkinson’s this advanced to have some assistance. Whether it be a fitness trainer or family member whatever they’re gonna just about have to.” (Spouse of participant, Lai et al., 2020) • “I think it’s a great idea, but I think it would be even better if I was not alone. Because I’m a people’s person, and I would enjoy having someone exercise with me.” (Participant, Lai et al., 2020)
	“Off periods,” comorbidities, fatigue	Patients discussed that comorbidities, fatigue, and “off” periods made it challenging to engage in exercise interventions: • “Since I’ve been diagnosed with PD and I felt low. . . I became less motivated to do anything in life. . . even when you invited me to do the exercises I felt apathetic.” (Participant, Khalil et al., 2017) • “I: What would you say was the most difficult or challenging thing that has come out of the physiotherapy treatment? P: It would be trying to do it every day. But that day, I know that if I do it I would be better and more flexible and more coordinated but it is just motivating myself to do it sometimes. It’s the hardest, that’s. . .it’s the hardest thing to do.” (Rowsell et al., 2020) • “I have a chronic problem in my knee and some of the balance exercises were causing me more pain. . . this did not stop me from doing the exercise. . . the therapist helped me in modifying the exercise so that it became more tolerable.” (Participant, Khalil et al., 2017) • “I lacked the habit of past exercise. This is the first time I have been in a structured program. When I first started, I used to feel tired even after performing only a few movements. This feeling, however, ceased off after few weeks.” (Participant, Khalil et al., 2017) • “And sometimes, you know with Parkinson’s you have your off periods where your drugs haven’t kicked in and there are times when I am having an off evening and I try and do my exercises, but the off spell seemed to be longer than normal, so I might have to miss it then, so it’s just choosing the time of day.” (Rowsell et al., 2020)
	Lack of outcome expectations	Negative expectations about intervention outcomes could act as a barrier to adherence: • “. . .but with a question mark. Am I in a phase that that [occupational therapy] can contribute?” (Patient, Sturkenboom et al., 2013) • “I feel I am physically better than other people. . . and the nature of my work requires a lot of movements. I work as a plumber; hence I move all the time” (Participant, Khalil et al., 2017)

*Objective 1: investigate PD patients’ and caregivers’ experiences of participating in clinical trials*

*Positive experiences of participating in research* (Table 2*; theme 1a*). Parkinson’s patients reported several factors linked to positive experiences of participating in clinical trials. The way in which interventions were delivered, and who was delivering them, was acknowledged as one such factor. In therapy trials (Daley et al., 2015; Sturkenboom et al., 2013), patients felt that the possession of specialist knowledge of Parkinson’s, honesty, and a nonjudgmental approach were crucial for building trust and engagement with the therapist. Participants in an occupational therapy trial described that a “click” with the therapist was an important facilitator to successful sessions (Sturkenboom et al., 2013), whereas in the trial of ballroom dancing, participants valued the instructors’ and researchers’ empathy and patience (Kunkel et al., 2017).

A recurring theme related to the positive effects of participation across several interventions was gaining self-confidence, which often stemmed from improving one’s physical function, such as increased mobility (Khalil et al., 2017; Kunkel et al., 2017; Lai et al., 2020; Paterson et al., 2005). This was closely linked to a sense of achievement resulting from being able to perform activities that were thought not to be possible before (Kunkel et al., 2017; Sturkenboom et al., 2013). However, participants noted that the benefits of interventions extended beyond physical gains and also included improvements in their mental well-being (Khalil et al., 2017; Paterson et al., 2005; Sturkenboom et al., 2013).

Participants enjoyed the social aspect of the research experience and appreciated the opportunity to meet new people (Kunkel et al., 2017; O'Brien et al., 2008). In trials of group interventions, friendships developed between participants who met socially and maintained contact after sessions (Kunkel et al., 2017; O'Brien et al., 2008). In a trial of group resistance training (O'Brien et al., 2008), some participants mentioned enjoying the experience due to the social aspect despite not noticing any physical improvements from the intervention.

Interacting with others who had Parkinson’s during the intervention sessions also facilitated a sense of belonging and was seen as a beneficial opportunity to exchange information related to the condition (Kunkel et al., 2017; O'Brien et al., 2008). This had a positive effect not only in relation to providing participants with a chance to share specific details about their symptoms or medication, but also offering a sense of comfort stemming from the knowledge that they are not the only ones affected by PD (O'Brien et al., 2008). Some participants indicated the importance of being in a group with people who were at a similar stage of the disease, as opposed to those whose Parkinson’s was more advanced (O'Brien et al., 2008). In the ballroom dancing trial, one partner also discussed the benefits of seeing how other couples coped with Parkinson’s (Kunkel et al., 2017).

Another positive aspect of trial participation identified by PD patients was being able to gain a better understanding of the condition as something that is individual to everyone (Rowsell et al., 2020; Sturkenboom et al., 2013). This provided a sense of comfort and helped the participants develop more efficient ways of managing their symptoms and coping with the disease.

*Assessment completion* (Table 2; *theme 1b*). The vast majority of data supporting this theme comes from the trial of therapeutic massage (Paterson et al., 2005), where PD patients were asked to complete the PDQ-39 (Parkinson’s Disease Questionnaire) and MYMOP (Measure Yourself Medical Outcome Profile). Generally, participants had no major problems with completing these assessments and reported only some minor issues, such as having difficulty indicating change without knowing previous scores. One patient observed that having the spouse involved in helping to complete the assessments may have influenced his responses.

The researchers noted several problems with the questionnaires, which seem to point to their limited ability to accurately reflect patients’ experiences (Paterson et al., 2005). For example: *“Choice of response was easy when something was very bad or very good*, *and most difficult ‘when it comes to*, *like dressing and that well if you can do part of it and not all of it.”* (Paterson et al., 2005) In addition, the answer options could not reflect that some patients adapted their lives or had someone else’s assistance in performing an activity so that it did not pose a practical problem, yet they were distressed due to not being able to do the activity by themselves (Paterson et al., 2005). Furthermore, the researchers identified the need for a “not applicable” option for activities that were not a part of patients’ daily life, such as looking after one’s home (Paterson et al., 2005). The questions on cognition also gave rise to a few problems: *“Two people had difficulties responding to ‘Had distressing dreams or hallucinations’ as they had hallucinations but they were not distressing”* (Paterson et al., 2005).

The most common criticism of the questionnaires was having difficulties selecting an accurate response due to the wording of the answer options, especially choosing between “occasionally” and “sometimes” (Paterson et al., 2005). The authors also noted that most participants required assistance with completing the questionnaires, such as having the questions read out for them (Paterson et al., 2005). Another patient had very little audible speech and needed his wife’s help to allow the interviewer to understand his answers (Paterson et al., 2005).

Participants and their partners also discussed that symptom fluctuations and medication timings could influence the assessments, leading to distorted results. For example, in the ballroom dancing trial (Kunkel et al., 2017), one spouse of participant with PD was worried that having a “bad day” at the time of final assessments of balance and mobility might result in the impact of the dance intervention not becoming evident. Similarly, a patient with PD from the same trial highlighted the variability of his PD symptoms from day-to-day and noted that he *“wasn’t feeling particularly right”* during his follow-up assessment, which negatively influenced his physical performance. In addition, some participants highlighted that daily fluctuations in their PD symptoms posed a challenge to averaging their impact out to one score over longer periods of time on the questionnaires (Paterson et al., 2005).

Furthermore, some participants indicated that the presence of comorbidities could make it difficult to distinguish between the impact of PD and other health problems on daily activities, as these effects often overlapped (Paterson et al., 2005). Several participants also noted being uncertain whether their cognitive deficits were caused by old age or PD.

*Objective 2: explore what motivates PD patients to enroll in clinical trials*

*Motivators* (Table 2; *theme 2a*). Parkinson’s patients gave a number of reasons for why they chose to participate in clinical trials. Direct personal benefit was often regarded as the main reason for enrolling in studies (Kim et al., 2015; Kunkel et al., 2017; O'Brien et al., 2008; Paterson et al., 2005; Rowsell et al., 2020). Participants perceived that trial interventions could slow down the progression of the disease and ameliorate its symptoms, with some patients expressing a willingness to try anything they could to get better.

On the other hand, some participants reported altruistic reasons for trial enrollment. This included a desire to contribute to science and wanting to benefit those who might suffer from PD in the future (Kim et al., 2015; Kunkel et al., 2017; O'Brien et al., 2008). In addition, some patients were equally motivated by both wanting to improve their symptoms of PD while also helping future patients (Kim et al., 2015). Participants also discussed the influence that other people could have in encouraging research participation (Kunkel et al., 2017; O'Brien et al., 2008; Sturkenboom et al., 2013). Some patients described feeling uncertain about getting involved in a study at first but later decided to enroll after talking to their friends and family (Kunkel et al., 2017). Other participants were encouraged by their caregivers (Kunkel et al., 2017; Sturkenboom et al., 2013) and doctors (O'Brien et al., 2008).

For some patients, the decision to participate in trials was motivated by wanting to socialize and have a regular activity to engage in (Kunkel et al., 2017; O'Brien et al., 2008). They described liking the prospect of *“having a routine twice a week*, *a trip out… and meeting people*, *socialising”* (Kunkel et al., 2017) and indicated this might be beneficial for their well-being (O'Brien et al., 2008). Other participants indicated they were motivated by their interest in the intervention (Kunkel et al., 2017; O'Brien et al., 2008; Rowsell et al., 2020). Some expressed they hoped to learn useful information and skills (Rowsell et al., 2020), whereas others wanted to find out more about the intervention (O'Brien et al., 2008) or *“thought it would be fun”* (Kunkel et al., 2017).

*Objective 3: Identify what are the enablers and barriers to participation in clinical trials from the perspectives of PD patients and their caregivers*

*Enablers* (Table 2; *theme 3a*). Participants and their caregivers noted several factors that positively affected their adherence to trial interventions. One such factor was ongoing supervision and support from the research team. For example, several patients who took part in exercise programs discussed that being monitored, either through a remote system or home visits, gave them a sense of accountability (Lai et al., 2020; Rowsell et al., 2020). This continuous supervision also allowed them to build the confidence to exercise and use any necessary technology (Khalil et al., 2017; Lai et al., 2020; Rowsell et al., 2020). In addition, encouragement from the coach or physiotherapist increased the participants’ motivation to exercise, enhancing adherence with the intervention (Khalil et al., 2017; Lai et al., 2020).

In some cases, positive attitudes toward trial design could be a facilitator to participation. Specifically, a few patients who took part in sham-controlled trials expressed that this design encouraged their enrollment (Kim et al., 2012). Similarly, openness toward the intervention could facilitate participation, for example, one caregiver from the occupational therapy trial expressed that they found it beneficial to let go of expectations and approach the intervention with an open mind (Sturkenboom et al., 2013).

Support and encouragement from the participant’s family, spouse, or caregiver were perceived as important factors influencing adherence (Daley et al., 2015; Khalil et al., 2017; Rowsell et al., 2020; Sturkenboom et al., 2013). This was prominent in physical exercise interventions, where partners and other family members motivated participants to exercise, sometimes even exercising with them (Khalil et al., 2017; Rowsell et al., 2020). Caregivers perceived their own involvement in the interventions and supporting their partners as natural (Sturkenboom et al., 2013). The involvement of caregivers was seen as beneficial by the participants and was related to practical factors, such as managing medication (Daley et al., 2015), as well as the caregivers gaining a better understanding of Parkinson’s (Sturkenboom et al., 2013).

Participants identified clear instructions as another factor promoting adherence to interventions. For example, patients in the trial of at-home exercise interventions appreciated that the exercises were *“simple and easy to follow”* (Khalil et al., 2017) and recognized it as a strong motivator to continue with the program. Similarly, participants who enrolled in the tele-monitored exercise intervention valued that the telehealth system *“was easy enough to operate and wasn’t obtrusive”* (Lai et al., 2020).

Patients with PD also appreciated the convenience of at-home interventions, identifying that it saves them time and travel costs (Lai et al., 2020; Mammen et al., 2018). In the trial of at-home exercise, participants also valued the flexibility of doing the exercises in their own time (Lai et al., 2020). In addition, both patients and caregivers in the trial of occupational therapy discussed the delivery of the intervention in their own homes (as opposed to a hospital) as more practical, highlighting that *“In the hospital you are in a theoretical situation*, *while my problems are here [at home]* (*…*)*”* (Sturkenboom et al., 2013). Similarly, participants perceived delivering adherence therapy face-to-face in their homes as essential and felt that other forms of delivery would not have the same effect (Daley et al., 2015). Travel arrangements and reimbursement were perceived as additional adherence facilitators (Khalil et al., 2017). Others appreciated accessible parking on site, and those who couldn’t drive, provision of a taxi (Kunkel et al., 2017).

*Barriers* (Table 2; *theme 3b*). Parkinson’s patients and their caregivers discussed several factors that served as barriers to participation in trials. For example, in the home-based exercise trial in Jordan, some early-stage patients still doubted their PD diagnosis (Khalil et al., 2017). Similarly, embarrassment or secrecy toward PD diagnosis due to cultural stigma was identified as another barrier to adherence (Khalil et al., 2017). Being physically active is not the norm in some cultures, especially for older people, and some participants reported feeling ashamed of exercising at home in front of their children and would have preferred traveling to a hospital for the training sessions (Khalil et al., 2017). In addition, some participants were unable to share their PD diagnosis with their families, including spouse and children. Therefore, they did not perform the exercises at home to avoid suspicion (Khalil et al., 2017).

In some cases, negative attitudes toward trial design could serve as a barrier to participation. Half of the participants who enrolled in sham-controlled surgery trials disclosed that the study design was a *“turn-off*, *a real big one”* (Kim et al., 2012) and mentioned feeling apprehensive or concerned. However, for the majority of participants, this initial reaction did not negatively influence their decision to participate (Kim et al., 2012).

Technology issues were identified as another barrier to intervention adherence. Participants in trials of online exercise (Lai et al., 2020) and virtual physician visits (Mammen et al., 2018) discussed that technical difficulties, such as Wi-Fi cutting off, were a source of frustration. Using the technology effectively by the participants required a learning curve, and some patients gave up after several unsuccessful attempts (Lai et al., 2020).

In line with frustration over technology issues, participants perceived lack of support and practical assistance during the initial stages of an intervention as an obstacle to adherence. Specifically, patients in the Internet exercise program discussed that they found the software “*a little difficult to get setup*” and expressed it would be beneficial to have some initial assistance (Lai et al., 2020). One spouse of a participant highlighted that support from a fitness trainer or family members is crucial in such interventions, especially for patients with advanced PD. Another participant expressed the preference for doing the exercises with somebody else present.

Furthermore, PD patients discussed that “off” times, comorbidities, and fatigue could make it challenging to engage in exercise programs (Khalil et al., 2017; Rowsell et al., 2020). Apathy and low motivation associated with PD also made it difficult to perform exercises regularly (Khalil et al., 2017; Rowsell et al., 2020). Some participants suffered from other health problems, which made certain exercises troublesome, whereas others described not being used to regular physical activity (Khalil et al., 2017). Another patient explained that at times due to experiencing “off” periods when the medication did not work optimally, they could not perform the exercises. However, they overcame this problem by finding an optimal time during the day to complete the training (Rowsell et al., 2020).

In some instances, participants’ negative expectations about the intervention outcomes could serve as a barrier to adherence. For example, one participant at an early stage of PD expressed he would not necessarily benefit from exercise, as he was still physically fit (Khalil et al., 2017). Another patient explained they approached occupational therapy *“with a question mark”*, as they were not convinced they were at a stage where this type of intervention would be helpful (Sturkenboom et al., 2013).

## Discussion

To our knowledge, this is the first systematic review on patient and caregiver experiences of participating in Parkinson’s disease clinical trials. Five key themes relating to experiences of participating in clinical trials, motivators for enrollment, as well as challenges and facilitators to participation were found. Consistent with previous research (Adler, Otado, & Kwagyan, 2020; Verheggen, Nieman, Reerink, & Kok, 1998), this review identified high patient satisfaction from participation in trials. The findings support and expand on the existing evidence on the importance of friendliness and expertise of research staff (Adler et al., 2020), trust built between researchers and participants (Watanabe et al., 2011), and social aspects of participation (Dobra, Guilmant, Higgins, & Fleming, 2017). The first theme, “positive experiences of participating in research,” demonstrated that social benefits extended beyond meeting new people by facilitating a sense of belonging and presenting an opportunity to share PD-related information. Our findings suggest that benefits of participation can expand beyond what is typically assessed by outcome measures, such as an increased understanding of PD and developing new ways of coping with the condition.

The second theme, “assessment completion,” demonstrated that participants had some problems with completing self-report questionnaires. These related mainly to having difficulty in choosing between response options (especially “occasionally” and “sometimes” on the PDQ-39) and requiring assistance from caregivers to complete them. These findings have implications for trial design, suggesting that attention needs to be paid to the support required by participants in order to complete these assessments. One suggestion proposed by Paterson and colleagues (2005) was to ensure that respondents could see the layout of the responses as a spectrum, in addition to having them read aloud. In addition, the researchers observed that the answer options of frequency of a problem often did not make logical sense, as the majority of patients experienced some difficulty all the time, rather than complete difficulty some part of the time (Paterson et al., 2005). However, this was not discussed with the participants during the interviews. It appeared that patients treated the response options as gradations of severity. Nevertheless, this may result in most responses falling in the middle range, potentially reducing sensitivity to change. Revision of the response options on the PDQ-39 may be therefore warranted. Another problem encountered by patients was having difficulty with distinguishing PD symptoms from other ailments, which is supported by previous research (Marinus, Ramaker, van Hilten, & Stiggelbout, 2002).

Consistent with existing evidence (Soule et al., 2016; Watanabe et al., 2011), our review demonstrated that participants’ enrollment in research is largely motivated by altruistic and health-related reasons. The theme “motivators” also highlighted that other people may play an important role in encouraging participation. This included caregivers, friends, and doctors or therapists. Moreover, the theme “enablers” underlined the importance of encouragement from healthcare workers for intervention adherence. Regular support and supervision from doctors and the research team should be considered in trial design as it gives patients a sense of accountability and allows them to develop self-confidence in relation to following the intervention. In addition, support from caregivers was perceived as beneficial. This is supported by previous research evaluating a PD psychoeducation program where the involvement of spouses and carers was viewed favorably by patients (A'Campo, Spliethoff-Kamminga, Macht, Edu Park, & Roos, 2010). Therefore, PD trials should aim to involve caregivers in the process as much as possible to increase adherence and retention. Many participants also appreciated the convenience of at-home or telehealth interventions, which contributed to adherence. Given the current pandemic climate, telehealth is in the process of expanding rapidly. However, our review identified that technological issues and lack of support served as barriers to adherence. Researchers should therefore consider ways to support older patient populations in operating technological equipment and incorporate remote methods to keep accountability.

### Quality of Included Studies

The main limitations of included papers from quality appraisal related to inconsistent transparency in reporting of the methodology, lack of critical examination of researchers’ roles and potential biases, and thin description of findings. Two studies were subject to moderator bias, as the interviewers were involved in delivering the interventions. This could have resulted in reluctance to report on negative experiences by the participants.

### Strengths and Limitations of the Review

Two reviewers independently carried out database search, critical evaluation of eligible papers, and data synthesis to ensure reliability and minimize bias. Study authors were directly contacted where information was missing. This review synthesized evidence from multiple trials, enabling the investigation of various interventions. However, this also warrants caution as the results may not be generalizable to all trials as there were no studies available for pharmacological intervention trials. Findings on patient and caregiver experiences were not separated due to little available data on caregivers’ perspectives. Limiting the database search to articles published in English and omitting gray literature may have excluded some relevant papers.

### Future Research

This review did not identify any eligible pharmacological trials. Qualitative studies that specifically investigate participant experiences in such trials are required to deepen our understanding of this issue. The majority of included studies (*n* = 9) were conducted in English-speaking developed countries. Some cultural issues were identified by this review. Further work is needed to explore trial participation experiences in this area. Few studies (*n* = 3) included participants’ caregivers. This review strengthened the evidence on the important role caregivers play in PD patients’ lives. Further research is therefore necessary to explore the support they provide to participants and their own experiences of trial participation. Only one study provided data on the experiences of questionnaire completion. Nonetheless, the findings are valuable and warrant further investigation in future research.

## Conclusion

This is the first systematic review on the experiences of patients with PD and their caregivers of participating in PD trials. Echoing previous findings (Adler et al., 2020; Dobra et al., 2017; Watanabe et al., 2011), positive experiences were guided by social interactions with other participants, expertise and empathy of research staff, and a sense of trust between patients and researchers. Benefits of participation extended beyond what is typically assessed by outcome measures and reflected gaining a better understanding of PD and developing new coping mechanisms for managing the condition. Parkinson’s patients may require additional support with completing self-report questionnaires, and the involvement of their caregivers in trials may be crucial to enable participation. Researchers must be aware of the important role of caregivers and should consider ways to involve them in research as much as possible.

## Supplementary Material

Archives_manuscript_acab083
