# Supplementary material for: Patient and Caregiver Experiences of Participating in Parkinson’s Disease Clinical Trials: A Systematic Review of Qualitative Studies
Source: Arch Clin Neuropsychol. 2021 Oct 20;37(3):654–76. doi: 10.1093/arclin/acab083 (PMC9035084; doi:10.1093/arclin/acab083)
Supplement: Archives_manuscript_acab083 [file archives_manuscript_acab083.docx]

**Supplementary data**

#

# Appendix 1: Search strategy

MEDLINE (via PubMed): Searched October 27, 2020

(((((((((((("parkinson disease"[MeSH Terms] OR ("parkinson"[All Fields] AND "disease"[All Fields])) OR "parkinson disease"[All Fields]) OR "parkinsons"[All Fields]) OR "parkinson"[All Fields]) OR "parkinson s"[All Fields]) OR "parkinsonian disorders"[MeSH Terms]) OR ("parkinsonian"[All Fields] AND "disorders"[All Fields])) OR "parkinsonian disorders"[All Fields]) OR "parkinsonism"[All Fields]) OR "parkinsonisms"[All Fields]) OR "parkinsons s"[All Fields]) AND ((("qualitative"[All Fields] OR "qualitatively"[All Fields]) OR "qualitatives"[All Fields]) OR (("interview"[Publication Type] OR "interviews as topic"[MeSH Terms]) OR "interview"[All Fields]))) AND ((((((("clinical trials as topic"[MeSH Terms] OR (("clinical"[All Fields] AND "trials"[All Fields]) AND "topic"[All Fields])) OR "clinical trials as topic"[All Fields]) OR "trial"[All Fields]) OR "trial s"[All Fields]) OR "trialed"[All Fields]) OR "trialing"[All Fields]) OR "trials"[All Fields])

**Appendix 2: Complete quality appraisal of included studies**

**Supplementary table 1**

*Quality appraisal of included studies*

|  | **1. Was there a clear statement of the aims of the research?** | **2. Is a qualitative methodology appropriate?** | **3. Was the research design appropriate to address the aims of the research?** | **4. Was the recruitment strategy appropriate to the aims of the research?** | **5. Was the data collected in a way that addressed the research issue?** | **6. Has the relationship between researcher and participants been adequately considered?** | **7. Have ethical issues been taken into consideration?** | **8. Was the data analysis sufficiently rigorous?** | **9. Is there a clear statement of findings?** | **10. How valuable is the research?** | **Comments** |
| --- | --- | --- | --- | --- | --- | --- | --- | --- | --- | --- | --- |
| **Daley et al. [16]** | Yes | Yes | Yes | Yes | Yes | No | Yes | Yes | Yes | This is the first study to evaluate experiences of receiving an adherence-promoting intervention in PD. The researchers discuss the contribution the study makes to existing knowledge and identify areas for future research. The findings are discussed in relation to other populations. Recommendations for future AT investigations are proposed. | Research aims are stated clearly, and the described methodology is appropriate. However, few elements of the study design are justified explicitly. Sufficient details surrounding recruitment strategy and data collection process are provided. The person who delivered AT also conducted the interviews. This hasn’t been critically examined in regard to potential bias and influence during data collection. Ethical approval was obtained from the research ethics committee and informed consent procedures were completed. There is an in-depth description of the analysis process and it is clear how themes were derived from the data. A secondary qualitative researcher naïve to AT and PD was involved in the analysis process. The findings are explicit, supported by quotes, and discussed in relation to the original research questions. Few negative experiences are identified, potentially due to moderator bias. |
| **Khalil et al. [23]** | Yes | Yes | Yes | Yes | Yes | No | Yes | Yes | Yes | The findings are discussed in relation to existing knowledge and directions for future research are proposed. The study provides valuable insight on sociocultural barriers to engaging in exercise interventions for people with PD in developing countries. | Whilst the aims of the study are explicit, reporting of the methods lacks some details and justification (e.g., the structure and setting of qualitative interviews are unspecified). Nevertheless, study aims and design appear compatible. Recruitment strategy is described in enough detail. There is no mention of relationship of interviewer to participant and researcher biases are not discussed. Ethical approval was granted by the research ethics committee and informed consent was obtained from the participants. Data analysis process is described in enough detail and it is explicit how the themes were derived from the data. The transcripts were coded by two independent researchers and respondent validation was used to increase credibility of findings. Additional contextual details aid understanding of findings. Nevertheless, some themes lack supporting quotes. |
| **Kim et al. [18]** | Yes | Yes | Yes | Yes | Yes | No | Yes | Yes | Yes | The findings are not discussed in relation to existing knowledge and directions for future research are not identified. However, the study informs the discussion on ethical issues surrounding sham-controlled trials by incorporating the perspectives of PD patients who actually participated in such trials. | Research aims are clear, and the design is appropriate. However, reporting of the data collection and analysis method lacks transparency, and few justifications are provided. The relationship between researchers and participants is not considered and researcher biases are not discussed. The study was deemed as exempt from US federal regulations; however, there is no mention of obtaining informed consent from participants. Qualitative findings are reported in a quantitative manner, and a small number of illustrative quotes is provided to support them. The findings are largely descriptive in nature and are not presented in depth. |
| **Kim et al. [17]** | Yes | Yes | Yes | Yes | Yes | No | Yes | Yes | Yes | The study provides insight into the phenomenon of therapeutic misconception in PD. There is some discussion of findings in relation to existing knowledge, but new areas where research is necessary  are not identified. | Although the aims, design and methods of the study are described in sufficient detail, few justifications are provided. As above, biases of the researchers and study design are not discussed. No background information regarding the interviewer(s) is provided. The article lacks an explicit statement on obtaining informed consent from participants. Data analysis process is described in enough detail for transparency. More than one analyst was involved in transcript coding and discrepancies were discussed in team meetings (peer checking). Qualitative findings are presented in a quantitative manner and few supporting quotes are included. The findings are for the most part descriptive in nature. |
| **Kunkel et al. [12]** | Yes | Yes | Yes | Yes | Yes | No | Yes | No | Can’t tell | The study demonstrates the feasibility of conducting a trial of ballroom dancing in people with PD and the researchers recommend a phase III trial. A separate report provides a detailed description of valuable qualitative data. However, these findings are not discussed in relation to existing literature. | Research aims are stated clearly and described methods appear to be consistent with the aims of the study. The researchers offer in-depth details on some aspects of the methodology, such as the intervention and recruitment. Justification is provided for many of the decisions made. However, the relationship between the researchers and participants has not been considered. Ethical approval for the study has been granted, but there is no clear mention of obtaining informed consent from the participants. Not enough detail is provided on the data analysis method to give transparency to the process. Results are discussed only in relation to feasibility and no qualitative findings are reported. However, detailed description of qualitative data collection, analysis and findings are presented in a separate data report obtained directly from the author. The report describes the findings in rich contextual detail with quotes provided in support. |
| **Lai et al. [24]** | Yes | Yes | Yes | Yes | Yes | Yes | Yes | Can’t tell | Yes | Qualitative findings are to some extent discussed in relation to existing knowledge. The authors make valuable recommendations for future trials of online exercise for people with PD. | Details surrounding study aims, recruitment, and data collection are presented clearly with justification provided. The relationship between the researcher and participants was critically examined (the interviewer was also a telecoach; this was identified as a potential cause of reluctancy of the participants to report negative feedback during interviews). Ethical approval was granted, and informed consent was obtained from the participants. Some details are provided on the data analysis process and theoretical frameworks are stated. However, the description of how the themes were derived from the data lacks transparency. It is also unclear how the applied theoretical frameworks were incorporated in the authors’ approach to analysis. Two independent researchers analysed the data. The description of qualitative findings is brief and lacks depth. Sufficient number of quotes are presented to illustrate each theme. |
| **Mammen et al. [19]** | Yes | Yes | Yes | Yes | Yes | No | Yes | Yes | Yes | This study provides valuable insight into factors underlying patients’ and physicians’ perceptions of virtual visits. The findings may be applicable to other conditions such Alzheimer’s disease or ALS. The findings are discussed in relation to existing literature and directions for future research are recommended. | The research aims are explicitly stated, and study design is compatible. Most elements of the study methods are reported clearly, with justification provided. Several measures undertaken to enhance validity are clearly stated. However, researcher biases are not discussed. Ethical approval was obtained, and informed consent procedures were completed. The analysis process is described in detail and illustrative quotes are presented to support the findings, with contradictory data taken into account. |
| **O’Brien et al. [20]** | Yes | Yes | Yes | Yes | Yes | Yes | Yes | Yes | Yes | The findings are discussed in the context of previous research and their implications are considered for other populations. The study grants insight into the perceptions and experiences of people with PD who took part in a resistance exercise programme.  Based on the results, the authors make recommendations for clinicians and researchers. | Most elements of the study design and methods are reported in detail and justified. The process of data collection is described with transparency and an interview schedule is provided. The background of each researcher is presented, and potential biases are explored. Ethical approval and informed consent were obtained. Data analysis process is described in great detail and methods used to enhance reliability are explicitly stated. Theoretical frameworks that underpin data analysis are reported and it is clear how they are incorporated in the process. In presenting the findings, numerous quotes are provided to support the derived themes. |
| **Paterson et al. [11]** | Yes | Yes | Yes | Yes | Yes | No | Yes | Yes | Yes | The study offers valuable insight into individual experiences of PD patients receiving therapeutic massage. The findings are discussed in relation to previous evidence and other patient populations with chronic progressive diseases. The authors make recommendations concerning the design of any future full-scale randomised trial in this area. The study explores participant views on some commonly used questionnaires in PD. | While the aims, design and methods of the study are generally described in detail, few justifications are provided. The methods and results of the study have been reported in full in a separate report. The role of the researchers and potential bias haven’t been critically examined. Ethical approval was granted, but there is no explicit statement on obtaining informed consent from the participants. Data triangulation and respondent validation have been employed within the study, but only one researcher analysed qualitative data. The results are largely descriptive and focus on the individual. Contextual details are provided in reporting. Some supporting quotes are present. The findings on questionnaires are in majority informed by researcher observations rather than participants’ voices. |
| **Rowsell et al. [21]** | Yes | Yes | Yes | Yes | Yes | Yes | Yes | Yes | Yes | This is the first study to explore expectations about participation in a home-based exercise programme for people with PD. The findings provide new insights into the role of technology and equipment in such programmes. The results are discussed in the context of previous research and carry implications for the design of future physical activity interventions for people with PD. | The description of the design and methods for this study is detailed in most areas, with justification provided for many of the decisions made. The researchers are reflexive in reporting of their own role, and the relationship between the interviewer and participants has been considered (the interviewer wasn’t previously involved in the intervention). Investigator triangulation (two analysts) and peer checking were utilised to enhance credibility of findings. Full ethics and governance approvals were received, and informed consent was obtained. Reporting of the findings is extensive and numerous quotes are presented to support the points made. |
| **Sturkenboom et al. [22]** | Yes | Yes | Yes | Yes | Yes | No | Yes | Yes | Yes | The study found that patients with PD and their caregivers report positive benefits from the home-based occupational therapy intervention, but its effectiveness is not yet ascertained. The positive perceived impact of occupational therapy warrants a large-scale trial. | Most elements of the study methods are clear, but few justifications are provided. Reporting of data collection and analysis methods lacks transparency. However, the research design appears to be consistent with the aims of the study. The interviews were conducted by a research assistant not involved in the randomized controlled trial. However, no further details are provided, and potential biases are not explored. Full ethical approval was granted, and informed consent was obtained. Triangulation was performed with data from interviews, question­naires and patient records. Reporting of qualitative findings is very brief and descriptive. Illustrative quotes are presented to support the derived themes. |
